# The grapevine R2R3-type MYB transcription factor VdMYB1 positively regulates defense responses by activating the *stilbene synthase gene 2* (*VdSTS2*)

**DOI:** 10.1186/s12870-019-1993-6

**Published:** 2019-11-07

**Authors:** Yihe Yu, Dalong Guo, Guirong Li, Yingjun Yang, Guohai Zhang, Shaohua Li, Zhenchang Liang

**Affiliations:** 1 College of Forestry, Henan University of Science and Technology, Luoyang, 471003 People’s Republic of China; 2 Beijing Key Laboratory of Grape Science and Enology and Key Laboratory of Plant Resource, Institute of Botany, the Chinese Academy of Sciences, Beijing, 100093 People’s Republic of China; 3 School of Horticulture Landscape Architecture, Henan Institute of Science and Technology, Xinxiang, 453003 People’s Republic of China

**Keywords:** Grapevine, MYB transcription factor, *VdMYB1*, Defense responses, Stilbene synthase, Powdery mildew

## Abstract

**Background:**

Resveratrol is a naturally occurring plant stilbene that exhibits a wide range of valuable biological and pharmacological properties. Although the beneficial effects of trans-resveratrol to human health and plant protection against fungal pathogens and abiotic stresses are well-established, yet little is known about the molecular mechanisms regulating stilbene biosynthesis in plant defense progress.

**Results:**

Here, we cloned and identified the Chinese wild grape (*Vitis davidii*) R2R3-MYB transcription factor VdMYB1*,* which activates defense responses against invading pathogen. *VdMYB1* transcripts were significantly upregulated after inoculation with the grapevine powdery mildew fungus *Erysiphe necator* (Schw.) Burr. Transient expression analysis using onion epidermal cells and *Arabidopsis thaliana* protoplasts showed that VdMYB1 was localized in the nucleus. Yeast one-hybrid assays revealed that VdMYB1 acts as a transcriptional activator. Grapevine leaves transiently overexpressing *VdMYB1* showed a lower number of fungal conidiophores compared with wild-type leaves. Overexpression of *VdMYB1* in grapevine leaves did not alter the expression of genes in salicylic acid- and jasmonate-dependent pathways, but affected the expression of stilbene synthase (*STS*) genes, key regulators of flavonoid metabolism. Results of electrophoretic mobility shift assays and in vivo transcriptional activation assays showed that VdMYB1 binds to the MYB binding site (MYBBS) in the *STS2* gene promoter, thus activating *STS2* transcription. In heterologous expression assays using tobacco leaves, VdMYB1 activated *STS2* gene expression and increased the accumulation of resveratrol.

**Conclusions:**

Our study showed that VdMYB1 activates STS2 gene expression to positively regulate defense responses, and increases the content of resveratrol in leaves.

**Electronic supplementary material:**

The online version of this article (10.1186/s12870-019-1993-6) contains supplementary material, which is available to authorized users.

## Background

Recognition of the invading pathogen is a key requirement for disease resistance. In plants recognition of specific pathogens activates chemical signaling, which trigger diverse cellular defense responses [1]. Many of these responses transmit the signals from the cell membrane to the nucleus, where these signals increases the transcript levels of several defense-related genes [2], which encode pathogenesis-related (PR) proteins and enzymes that produce antimicrobial secondary metabolites [3]. Thus, transcriptional regulation of plant defense-related genes play a critical role in defense responses and disease resistance.

Transcription factors (TFs) bind to specific promoters, and activate the expression of downstream genes. In *Arabidopsis thaliana*, plant defense responses involve the activation of more than 1500 TFs belonging to six major families, including ERF, TGA, WRKY, Whirly, NAC (NAM, ATAF, and CUC), and R2R3 MYB. The ERF family TFs bind to the GCC-box, which is found in the promoters of several *PR* genes [4]. Genomic targets of TGA TFs include *PR* genes and the gene encoding glutathione S-transferase [3]. WRKY TFs bind the W-box elements in *NON-EXPRESSOR OF PR1* promoter and other *PR* gene promoters. Whirly TFs bind to the PB element in the *PR-10a* gene promoter [5]. NAC TFs target the promoter of *EARLY RESPONSIVE TO DEHYDRATION*, and activate the expression of defense genes [6].

The MYB superfamily is the largest known family of plant TFs, containing approximately 198 members in *Arabidopsis* and 183 in rice (*Oryza sativa*) [7, 8]. According to the number of conserved SWI3, ADA2, N-CoR, and TFIIIB (SANT) domains, The MYB TFs are divided into three subfamilies: MYB-related TFs (one SANT domain), R2R3-type TFs (two SANT domains), and R1R2R3-type TFs (three SANT domains) [8]. The MYB proteins exhibit diverse functions in plant development, biotic and abiotic stress responses, primary and secondary metabolism, hormone synthesis, and signal transduction [9]. Most of the R2R3-type MYB TFs function in the response to biotic and abiotic stresses. In *Arabidopsis*, AtMYB2, AtMYB21, AtMYB24, AtMYB30, AtMYB96, AtMYB102, and AtMYB108 function in stresses included by cold, wounding, salinity, drought, and pathogen infection [7]. Moreover, *PnMYB134* in *Populus nigra* was found in response to biotrophic rust fungus, MYB134 overexpressing and silencing lines accumulated higher and lower amounts of flavan-3-ols, respectively, resulting in altered pathogen infection [10]. In grapevine (*Vitis spp.*), R2R3-type MYB proteins play positive or negative roles in the production of enzymes involved in the biosynthesis of flavonoids and phenylpropanoids [11–13]. However, few documents have reported that the MYBs regulating stilbenes are involved in grapevine defense response.

Grapevine is the most economically important tree fruit crop in the world [14, 15]. The most commonly cultivated species of grapevine, *Vitis vinifera*, is highly susceptible to the fungal pathogen *Erysiphe necator* (Schw.) Burr. [16]. The resulting powdery mildew (PM) disease reduces fruit yield and wine quality. Therefore, identification of genetic sources of resistance to PM will be useful for grapevine breeders [17]. China is a major center of origin of *Vitis* species, and some Chinese wild *Vitis* species show strong resistance to major pathogen, including pathogens of cultivated *Vitis* species [17]. For example, the Chinese wild grapevine (*V. davidii*) shows strong resistance to several fungal pathogens, including *E. necator* [17]. Although several disease resistance genes have been cloned from the Chinese wild grapevine *V. pseudoreticulata*, disease resistance genes in *V. davidii* are yet to be examined.

To explore the function of MYB family TFs in defense responses, we identified and cloned the *MYB1* gene from *V. davidii* (*VdMYB1*). We show that *VdMYB1* transcripts respond to pathogen infection, and VdMYB1 TF regulates the defense response by activating the stilbene synthase 2 (*STS2*) gene, which encodes a key enzyme in the stilbenoids biosynthesis pathway. Thus, our findings suggest that *VdMYB1* play a key role in the defense response of grapevine to invading pathogens.

## Results

### Identification of VdMYB1

**Fig. 1:**
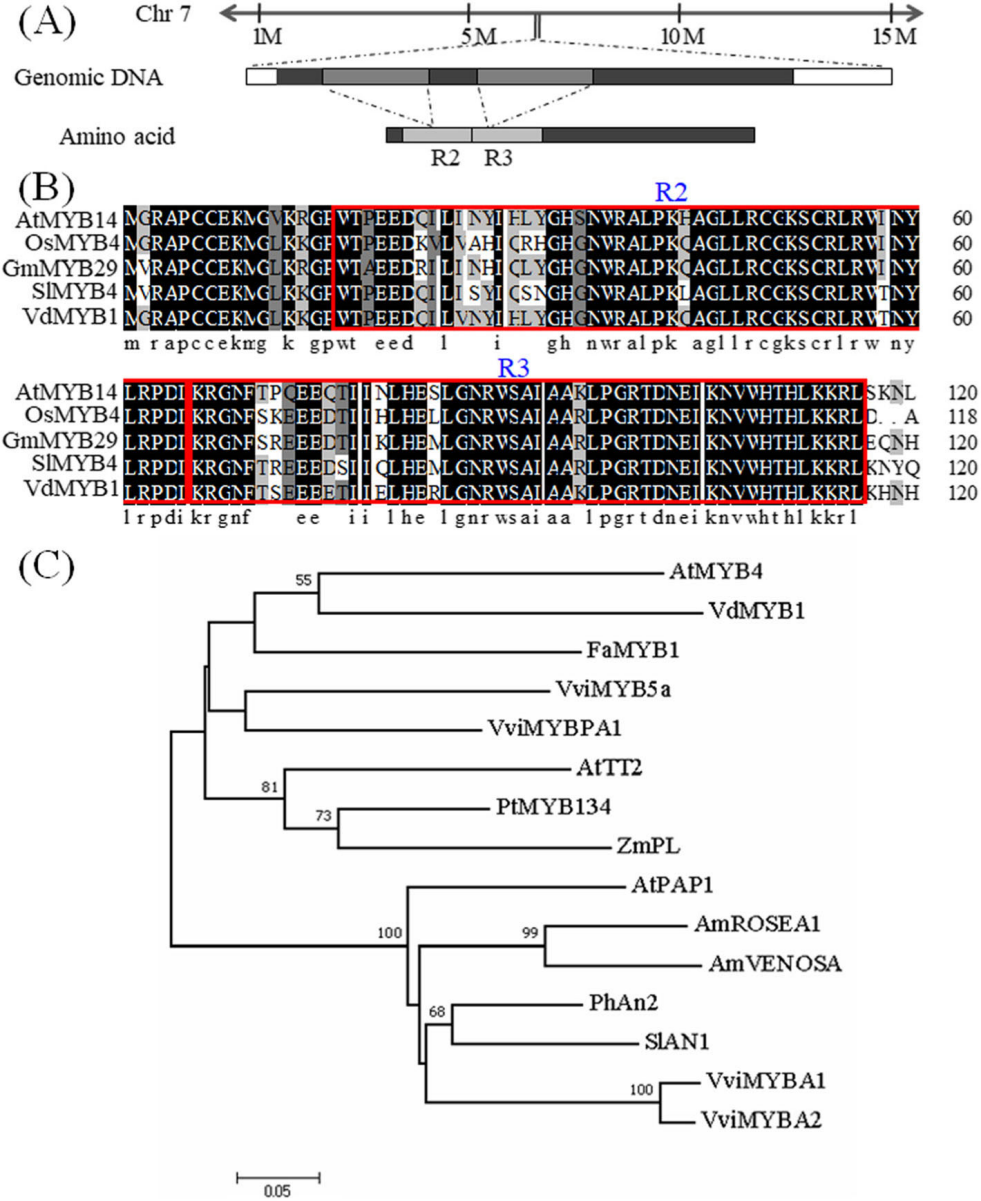
Sequence analysis of *VdMYB1*. **a** Schematic representation of the *VdMYB1* gene and cDNA structure, and the chromosomal location of *VdMYB1*. **b** Multiple sequence alignment of the R2R3-type motifs in MYB TFs from different plant species. **c** Phylogenetic analysis of VdMYB1 proteins with selected R2R3 MYB proteins from other species. The phylogenetic tree R2R3 MYB full-length was constructed using MEGA 7.0.26 [18] using Neighbor joining methods with p distance model and 1000 bootstrap replicates. Bootstrap values higher than 50% are shown. SlAN1 (*Solanum lycopersicum* AN1, AAQ55181), PhAN2 (*Petunia × hybrida* AN2, AAF66727), VviMYBA1 (*Vitis vinifera* MYBA1, BAD18977), VviMYBA2 (*V. vinifera* MYBA2, BAD18978), AtPAP1 (*Arabidopsis thaliana* PAP1/MYB75, AAG42001), AmVENOSA (*Antirrhinum majus* VENOSA, ABB83828), AmROSEA1 (*A. majus* ROSEA1, ABB83826), ZmPL (*Zea mays* PL, AAB67721), FaMYB1 (*Fragaria × ananassa* MYB1, AAK84064), AtMYB4 (*Arabidopsis* MYB4, NP_850879), VviMYB5a (*V. vinifera* MYB5a, AAS68190), VviMYBPA1 (*V. vinifera* MYBPA1, AM259485)

Full-length cDNA of *VdMYB1* was obtained by rapid amplification of cDNA ends (RACE) -PCR (GenBank accession: MK188872). Alignment of the genomic DNA of *VdMYB1* with the whole genome sequence of *V. vinifera* cultivar Pinot Noir showed that *VdMYB1* is located on chromosome 7 (Fig. 1a). By aligning the genomic and cDNA sequences of *VdMYB1*, we found that *VdMYB1* contains two introns and three exons (Fig. 1a). The *VdMYB1* open reading frame (ORF) encodes a predicted protein of 272 amino acids, with a theoretical isoelectric point (*pI*) of 6.51 and a deduced molecular mass of 30.437 kDa. Analysis using SMART and PROSITE programs revealed an R2R3-type MYB domain at the N-terminus of VdMYB1 (Fig. 1a, b). Phylogenetic analysis showed that the R2R3-type MYB genes from related species clustered into two subgroups (Fig. 1c).

### *VdMYB1* expression is induced by various defense signals

**Fig. 2:**
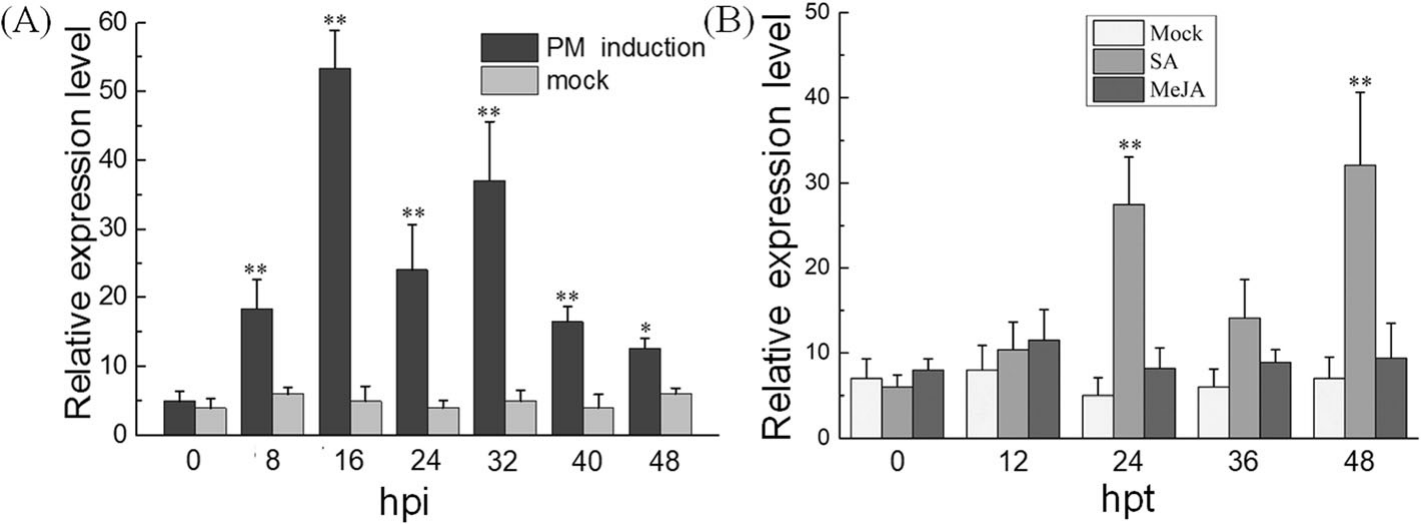
Expression analysis of *VdMYB1* in grapevine leaves treated with various defense signals. **a** Transcripts of *VdMYB1* were measured in detached leaves of *V. davidii* cv. Tangwei infected with *E. necator* using qRT-PCR. **b** Expression profiles of VdMYB1 in response to exogenous plant defense signaling molecules (SA, MeJA) in the leaves of *V. davidii*. cv. Tangwei. Leaves sprayed with sterile water were used as a mock treatment. Leaves were collected at different time points as indicated (hpi, hours post-inoculation; hpt, hours post treatment). The *VdGAPDH* gene was used as an internal control. Error bars represent standard deviation (SD). Asterisks indicate statistically significant differences (*P < 0.05, **P < 0.01; Student’s *t*-test)

To investigate whether *VdMYB1* is involved in defense responses, we analyzed *VdMYB1* transcript levels in detached leaves of *V. davidii* cv. Tangwei after inoculation with PM pathogen *E. necator* using quantitative real-time PCR (qRT-PCR). The level of *VdMYB1* transcripts was significantly increased in response to PM infection, as soon as 8 h post-inoculation (hpi), reaching a peak at 16 hpi, and *VdMYB1* expression was maintained at a high level until 24–48 hpi (Fig. 2a). To determine whether *VdMYB1* also responds to plant hormones, *V. davidii* cv. Tangwei leaves were treated with SA, methyl jasmonate (MeJA), and the *VdMYB1* expression level was investigated by qRT-PCR. Results showed that *VdMYB1* transcripts can quickly response to SA, and the expression level reached the first peak at 24 h post treatment (hpt), and declined at 36 hpt, then surged to its second peak at 48 hpt. However, *VdMYB1* transcripts showed no significant change under MeJA treatment (Fig. 2b).

### *VdMYB1* encodes a functional transcription factor

**Fig. 3:**
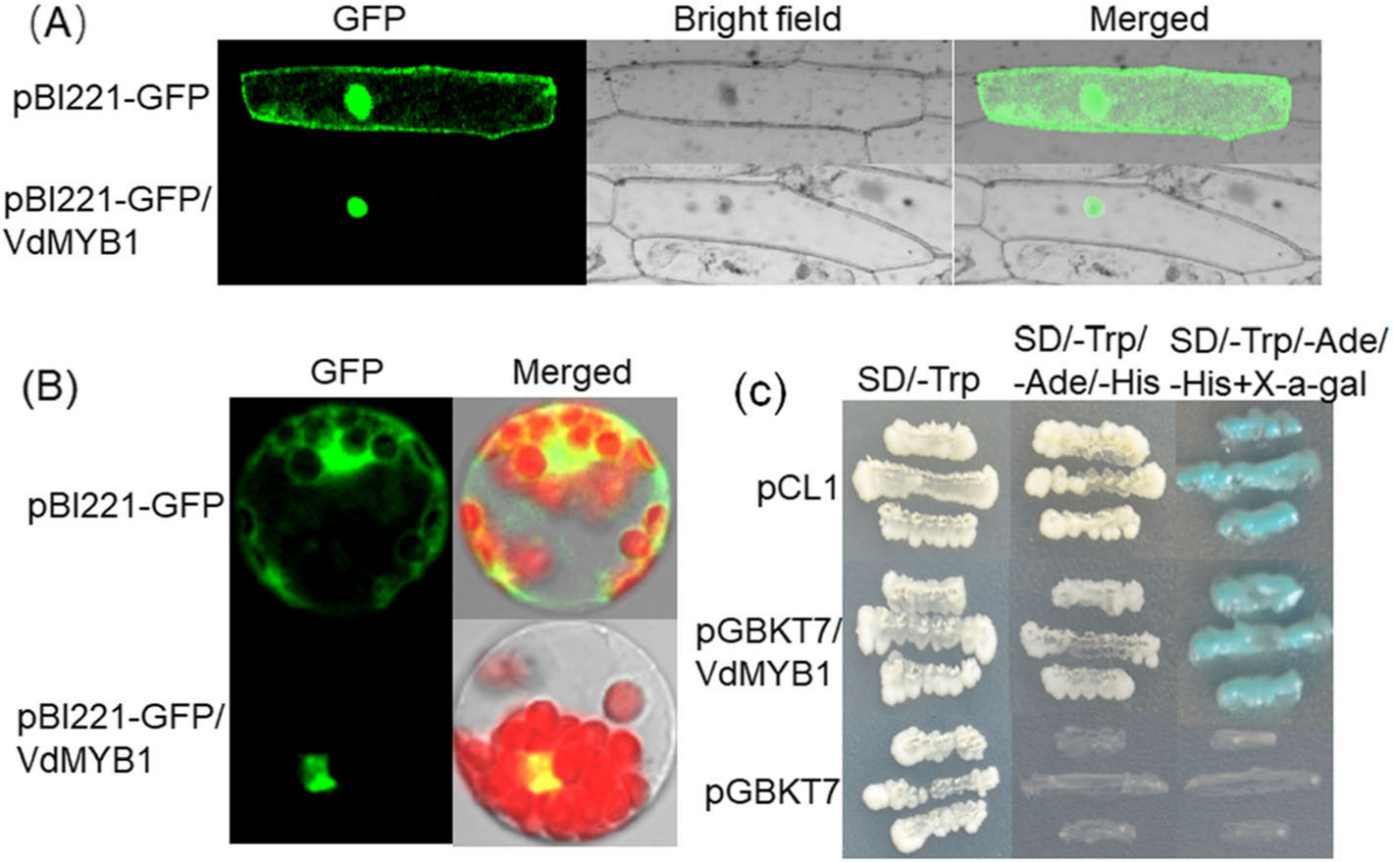
Subcellular localization and transcriptional activation function of *VdMYB1*. *VdMYB1* expression in onion epidermal cells (**a**) and *Arabidopsis* protoplasts (**b**). The expression of *VdMYB1*-GFP fusion protein was detected usingconfocal laser -scanning microscopy 16 h after transformation. **c** Transcriptional activation function of VdMYB1. The constructs pGBKT7/*VdMYB1*, pCL1 (positive control) and pGBKT7 (empty vector; negative control) were expressed in the yeast strain AH109. Transformants were incubated on SD/−Trp, SD/−Trp/−His/−Ade, and SD/−Trp/−His/−Ade/+X-*α*-gal media, and the growth of yeast cell and t*β*-galactosidase activity were measured

To study the subcellular localization of VdMYB1, we generated a construct encoding a fusion of VdMYB1 with the green fluorescent protein (GFP) under the control of the cauliflower mosaic virus (CaMV) 35S promoter. Introduction of the construct into onion epidermal cells and Arabidopsis protoplasts revealed GFP signal in the nuclei (Fig. 3) By contrast, introduction of the GFP gene alone under the control of CaMV 35S promoter into onion epidermal cells showed fluorescence throughout the cell (Fig. 3a). In *Arabidopsis* protoplasts, the VdMYB1-GFP fusion protein was also localized to the nucleus (Fig. 3b). Together, these observations indicate that VdMYB1 localizes to the nucleus.

To test whether VdMYB1 functions as a transcriptional activator, we fused the VdMYB1 ORF to the GAL4 DNA binding domain in the pGBKT7 vector, and introduced the construct into yeast (*Saccharomyces cerevisiae*) strain AH109. Transformed yeast cells harboring the pCL construct (expressing full-length GAL4), pGBKT7 empty vector, or pGBKT7/VdMYB1 grew well on synthetic defined (SD) medium lacking tryptophan (SD/−Trp) (Fig. 3c). Yeast cells transformed with pGBKT7/VdMYB1 or pCL grew better on SD medium lacking Trp, adenine (−Ade), and histidine (−His) (SD/−Trp-Ade-His), and also showed *β*-galactosidase activity (Fig. 3c). Cells harboring the negative control plasmid pGBKT7 showed no growth on SD/−Trp-Ade-His medium and no *β*-galactosidase activity (Fig. 3c). These data suggest that the R2R3-type MYB protein VdMYB1 functions as a transcriptional activator in yeast.

### Transient expression of *VdMYB1* in grapevine affects defense responses

**Fig. 4:**
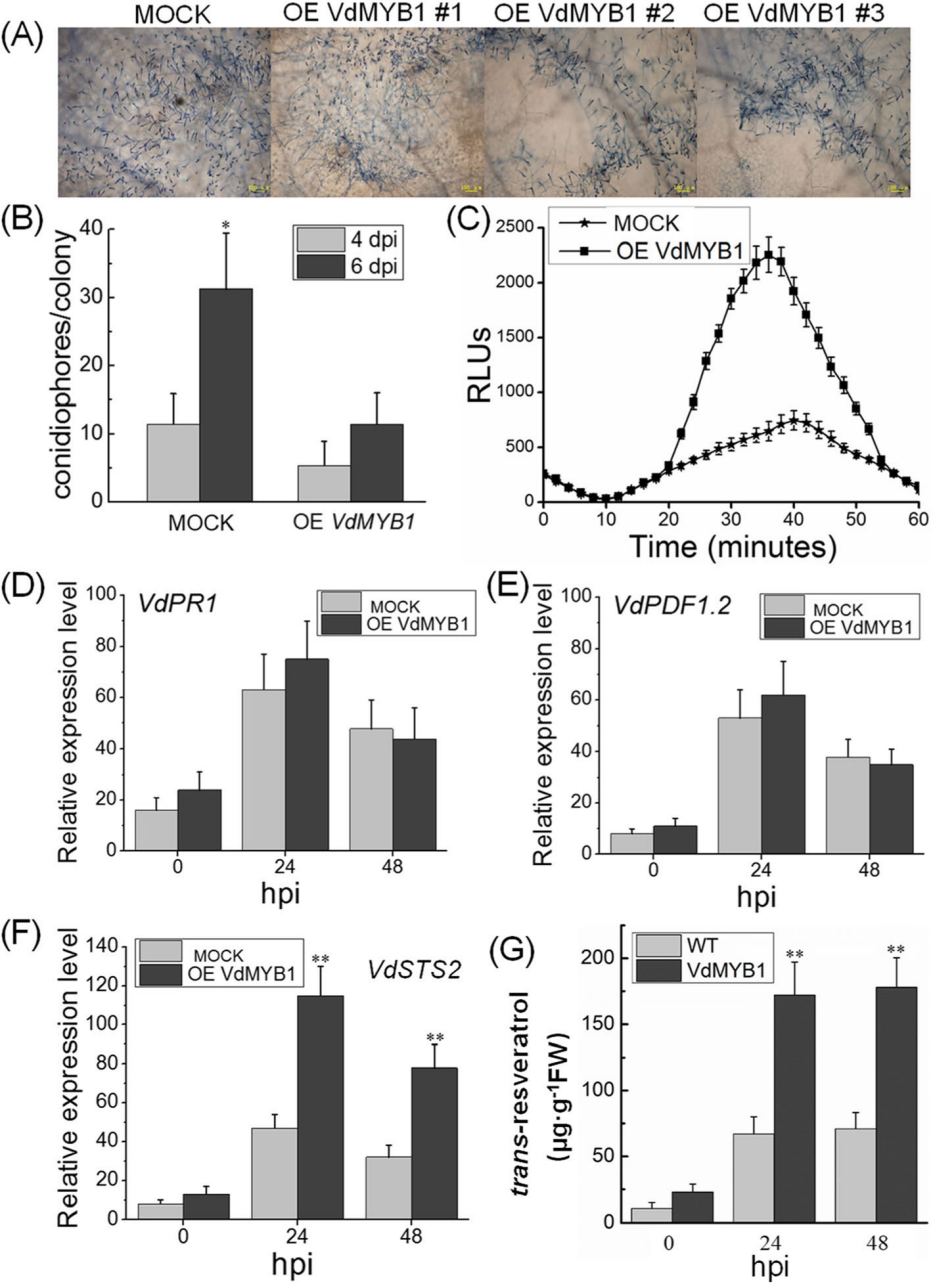
Transient expression of *VdMYB1* in grapevine leaves affects defense responses. **a** Trypan blue stained grapevine leaves inoculated with *E. necator* at 6 dpi. OE VdMYB1 #1, OE VdMYB1 #2, OE VdMYB1 #3 represent three independent replicates. **b** Number of conidiophores per colony of *E. necator* on grapevine leaves at 4 and 6 dpi. This method was used to quantify fungal growth on grapevine leaves. Error bars represent SD of three independent biological replicates (*n* = 30). **c** Kinetics of reactive oxygen species (ROS) production monitored over 60 min in leaf challenge with *E. necator*. Error bars represent SD of three biological replicates. **d**-**f** Expression of defense-related genes in VdMYB1-overexpressing leaves and mock leaves. Relative transcript levels of *VdPR1*, *VdPDF1.2*, and *VdSTS2* were examined at various times using qRT-PCR. The *VdGAPDH* gene was used as an internal control. Error bars represent SD of three biological replicates per genotype and time -point. Hpi, hours post inoculation. **g** Measurement of *trans*-resveratrol content in mock and *VdMYB1*-overexpressing leaves after pathogen infection using high- performance liquid chromatography (HPLC). Mock: leaves infiltrated with *Agrobacterium* carrying no vector. The data represent means ± SD of three independent experiments. Asterisks indicate statistically significant differences (*P < 0.05, **P < 0.01)

To examine the function of *VdMYB1* in plant defense, *VdMYB1* was transiently overexpressed in grapevine leaves using the agroinfiltration method. Leaves infiltrated with *Agrobacterium tumefaciens* cells not carrying any vector served as a mock treatment. Approximately 24 h after transformation, *VdMYB1*-overexpressing leaves and mock leaves were inoculated with *E. necator* at 6 day post-inoculation (dpi). Trypan blue staining revealed greater development of PM hyphae on mock leaves than on *VdMYB1-*overexpressing leaves (Fig. 4a). Next, to quantify fungal reproduction, we determined the number of conidiophores on leaves at 4 and 6 dpi (Fig. 4b). The *VdMYB1* overexpressing leaves showed fewer conidiophores than mock leaves both at 4and 6 dpi (Fig. 4b). After PM infection, fungal reproduction was much faster on mock leaves than on *VdMYB1*-overexpressing leaves (Fig. 4b). Since reactive oxygen species (ROS) play a major role in plant defense against pathogen attack, we analyzed the level of ROS in *VdMYB1*-overexpressing leaves and mock leaves after inoculation with *E. necator*. We found that the ROS were strongly induced at 20 min after inoculation in *VdMYB1*-overexpressing leaves, which reached the peak at 38 mi (Fig. 4c). By contrast, mock leaves did not show crease in the level of ROS after inoculation with *E. necator* (Fig. 4c). These findings indicate that *VdMYB1* enhances resistance to fungal pathogens in grapevine leaves.

Next, we investigated whether transient overexpression of *VdMYB1* in grapevine leaves affects transcript levels of defense-related genes. We first monitored transcript levels of *VdMYB1* in, three independently transformed leaves showed higher expression level than mock in difference time point (Additional file 2: Figure S1). After challenging the leaves with *E. necator, VdMYB* transcripts were upregulated (Additional file 2: Figure S1). Salicylic acid-dependent gene *PR1* and jasmonate-dependent gene *PDF1.2* was also detected. Results showed that there was no difference in *VdMYB1* expression level between *VdMYB1*-overexpressing leaves and mock leaves (Fig. 4d, e).

In grapevine, STS enzymes mediate the biosynthesis of stilbenes and exhibit broad-spectrum resistance to various pathogens [19]. To explore the molecular mechanisms of *V. davidii* cv. Tangwei resistance to PM, transcriptome sequencing was preformed after challenge with PM. Among of these 10 candidate genes, only one encode stilbene synthase, which showed high homolog with *VvSTS2* (GenBank accession no. XM_003634020) (data not published). To investigate whether VdMYB1 regulate *VdSTS2*, we measured the expression of *VdSTS2* in *VdMYB1*-overexpressing leaves and mock leaves. Transcripts of *VdSTS2* increased rapidly following inoculation with *E. necator*, and peaked at 24 h both in *VdMYB1*-overexpressing leaves and mock leaves, although *VdSTS2* transcripts were more abundant in transgenic *VdMYB1*-overexpressing leaves than in the mock leaves at all time points (Fig. 4f). Additionally, resveratrol contents in *VdMYB1*-overexpressing leaves was higher than in mock leaves at 24 and 48 dpi (Fig. 4g). Based on these data, we speculate that VdMYB1 TF activates the expression of *VdSTS2*.

### VdMYB1 targets the STS2 gene promoter

**Fig. 5:**
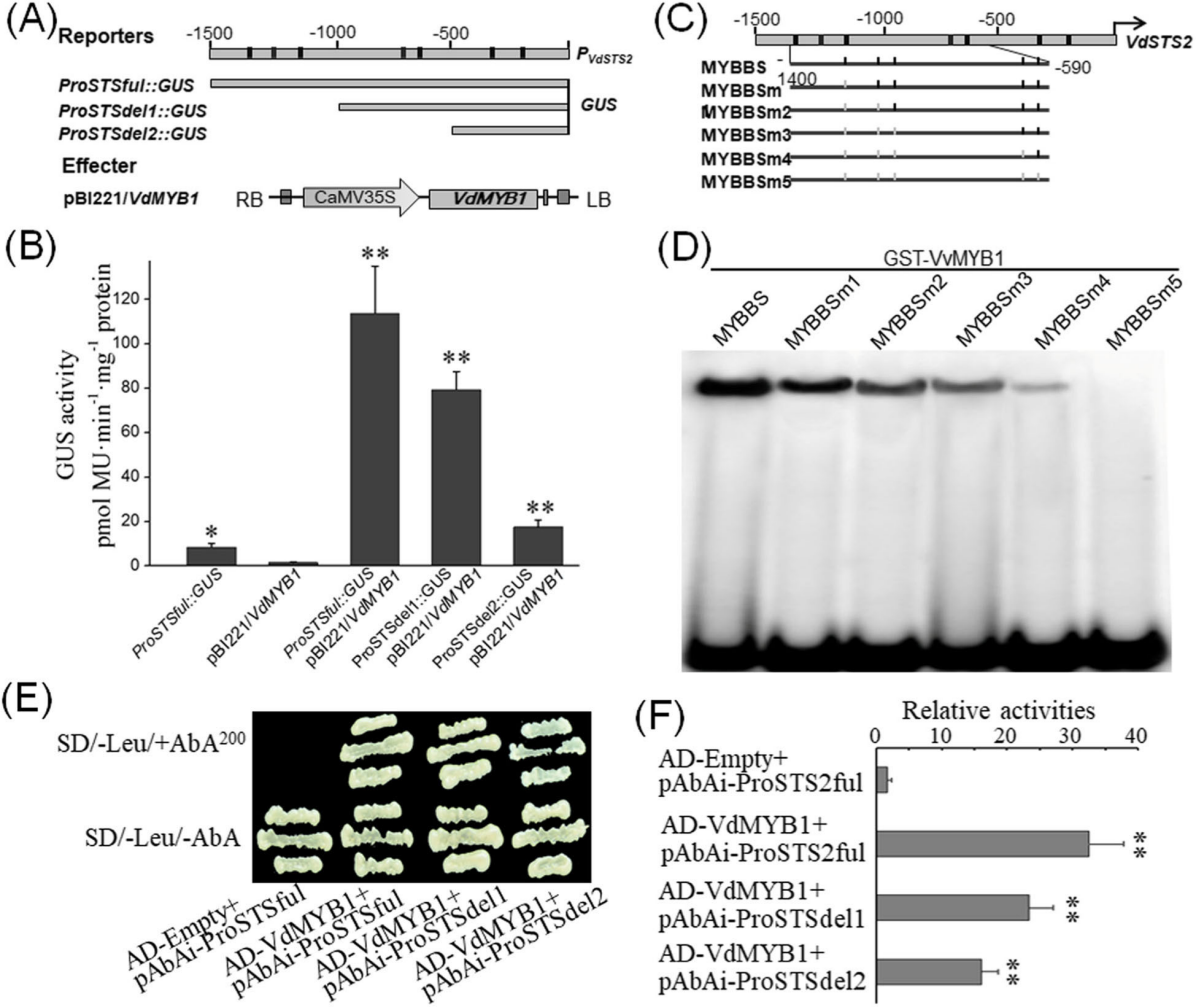
VdMYB1 binds to the promoter of *VdSTS2.* **a** Schematic representation of reporter and effector constructs. Deletion variants of the *VdSTS2* promoter were cloned into the pC0390GUS vector to drive the expression of *GUS* reporter. The pC0390GUS empty vector was used as a negative control. The full-length cDNA of *VdMYB1* was cloned into pBI221 vector to serve as an effector. **b** Measurement of GUS activity in *Arabidopsis* leaf protoplasts after the co-transformation of reporter and effector constructs. Samples were analyzed at 24 h after co-transformation. Error bars indicate SD of three independent experiments. **c** Schematic representation of the mutated MYB TF binding sites (MBSBSs) in the *VdSTS2* gene promoter. **d** EMSA showing that VdMYB1 specifically binds to the *VdSTS2* promoter. Purified GST-VdMYB1 protein was incubated with ^32^P-labeled DNA probes (*STS2* promoter fragments) and subjected to EMSA using polyacrylamide gel electrophoresis (PAGE). Competition for the formation of protein-DNA complex was performed using 50X unlabeled probes. **e** Analysis of the transcriptional activation function of *VdMYB1* using yeast one-hybrid (Y1H) assay. Transformed yeast cells were examined by the growth performance on SD/−Leu supplemented with 200 mg/L of AbA. The GAL4 activation domain (AD) empty vector and pAbAi-proVdSTS2ful vector were used as negative controls. **f** Activity of *β*-galactosidase transformed yeast cells as described in (**e**). Statistical significance was determined using Student’s t-test in different samples (*, *P* < 0.01; **, *P* < 0.001)

To confirm whether *VdMYB1* functions as a TF and targets the promoter of *VdSTS2*, we conducted in vitro and in vivo assays. Based on the reference genome sequence of *V. vinifera* cv. Pinot Noir, *VdSTS2* promoter region (− 1500–0 bp) was cloned by the homolog clone method. Analysis of the *VdSTS2* promoter sequence revealed numerous MYB TF binding sites (MYBBSs) (Fig. 5a). To investigate whether *VdMYB1* targets the promoter of *VdSTS2* in vitro, we generated a set of serial deletions of the *VdSTS2* promoter, and fused these with the *GUS* reporter gene (Fig. 5a). We also constructed a *VdMYB1* overexpression vector as an effector. We then co-transformed the effector and reporter constructs into *Arabidopsis* leaf protoplasts and measured GUS activity as an indicator of promoter activation. Constructs *ProSTSful* and *ProSTSdel1* containing full-length and deleted variant of the *VdSTS2* promoter, respectively, showed higher GUS activity than the *ProSTSdel2* construct, which lacked the sequence from --1500 to 500 bp (Fig. 5b). These results indicate that *VdMYB1* directly targets the *VdSTS2* promoter between − 1500 to − 500 bp.

To further investigate whether *VdMYB1* directly binds to the *VdSTS2* promoter between − 1500 to − 500 bp in vitro, we performed electrophoretic mobility shift assays (EMSA) with recombinant proteins and nine overlapping oligonucleotide probes encompassing the region from − 1400 to -590 bp of the *VdSTS2* promoter (Fig. 5c). The recombinant GST-VdMYB1 protein bound probes containing MYBBSs, but did not bind the probe containing zero MYBBSs, the binding activity of the recombinant protein was correlated with the number of MYBBSs (Fig. 5d). To further verify the transcriptional activation function of *VdMYB1*, we performed yeast one-hybrid (Y1H) assays with the *VdSTS2* gene promoter. Results of Y1H showed that the yeast strain with the bait reporter gene driven by the *VdSTS2* promoter grew well on media containing the AbAi antibiotic when co-transformed with the *VdMYB1* gene (Fig. 5e). These results show that VdMYB1 function as an R2R3-type MYB transcriptional activator, which binds to the MBSBS in the *VdSTS2* promoter and activates the transcription of *VdSTS2* gene in grapevine.

### VdMYB1 enhances disease resistance by activating the *VdSTS2* gene

**Fig. 6:**
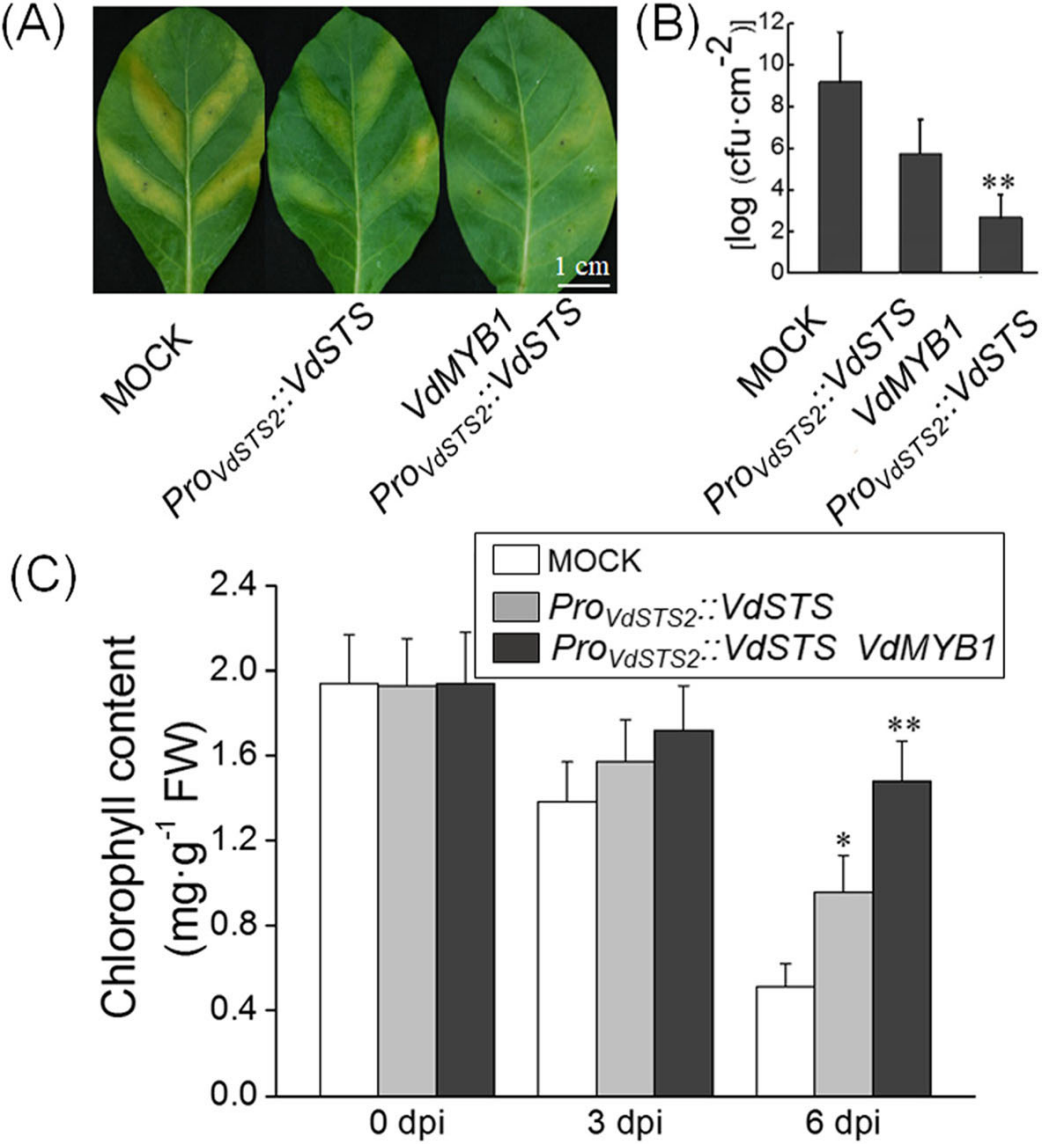
Transient expression of *VdMYB1* in tobacco leaves enhances disease resistance. **a** Transiently transformed tobacco leaves show enhanced resistant against *R. solanacearum*. Leaves were infiltrated with *R. solanacearum* (1 × 10^7^ cfu/ml), and disease symptoms were checked at 6 dpi. **b** Estimation of bacterial population in leaves transformed with *Agrobacterium* containing overexpression vector or no vector (mock treatment) **c** Chlorophyll content was measured in leaves transformed with Agrobacterium containing overexpression vector or no vector (mock treatment) Error bars represent SD. Asterisks indicate statistically significant differences (*P < 0.05, **P < 0.01; Student’s t-test)

**Fig. 7:**
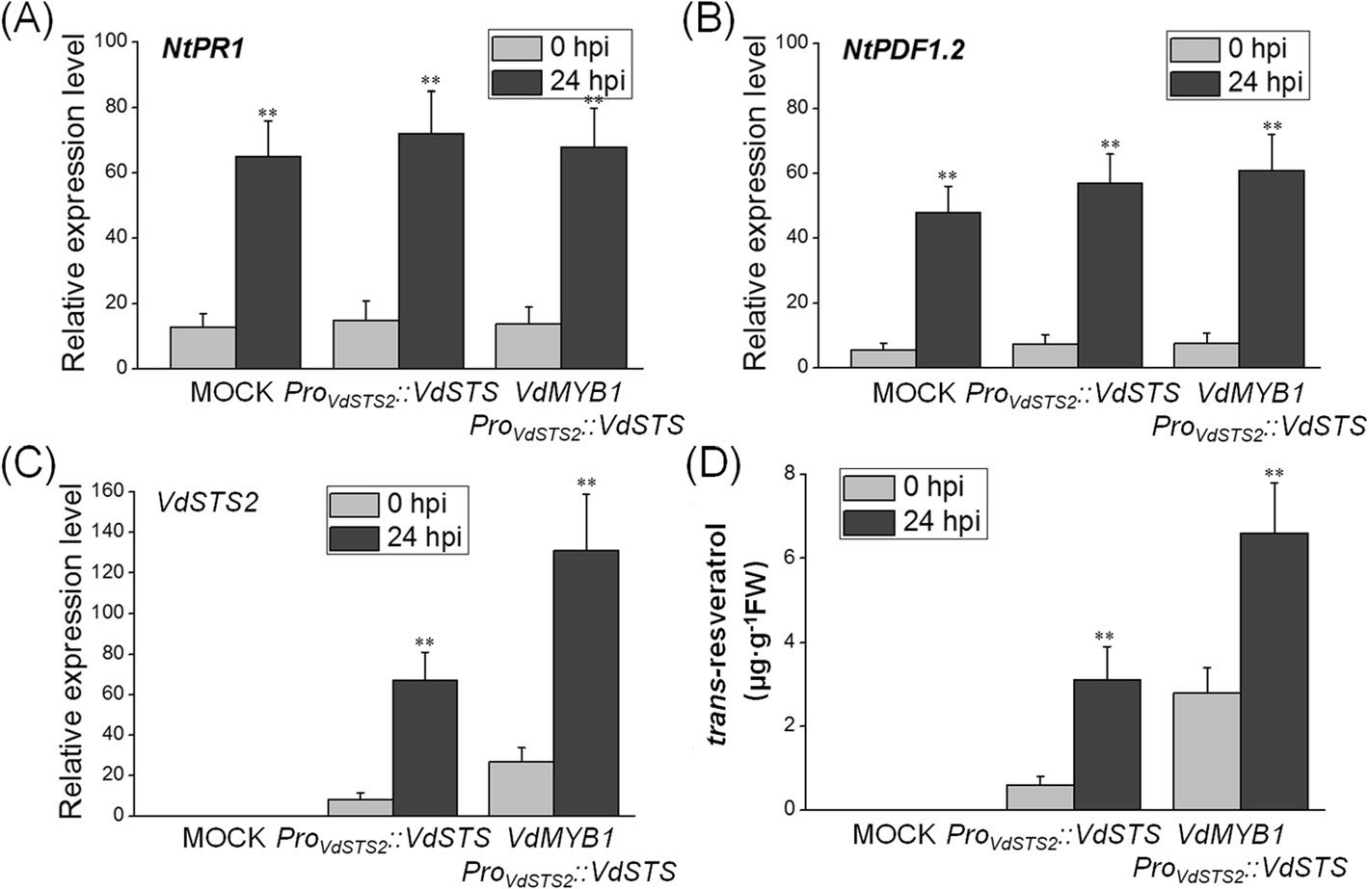
Effect of *VdMYB1* expression on defense-related gene expression and resveratrol content tobacco in tobacco leaves. **a**-**c** Expression of defense-related genes in tobacco leaves transformed with *Agrobacterium* containing overexpression *VdSTS2* (or *VdMYB1*) or mock after pathogen infection. Relative expression levels of *NtPR1*, *NtPDF1.2*, and *VdSTS2* were examined at 0 and 24 hpi using qRT- PCR. *NtEF1α* gene was used as an internal control. Hpi, hours post inoculation. **d** Measurement of *trans*-resveratrol content in mock and overexpression *VdSTS2* (or *VdMYB1*) leaves after pathogen infection using HPLC. Error bars represent SD. Asterisks indicate statically significant differences (*P < 0.05, **P < 0.01; Student’s *t*-test)

Next, we performed transient expression assay in tobacco (*Nicotiana tabacum*) leaves to verify that *VdMYB1* activates *VdSTS2* expression in a heterologous system. The expression of *VdSTS2* under the control of its native promoter enhanced resistance against to *Ralstonia solanacearum* compared with the mock treatment (grapevine leaves infiltrated with *Agrobacterium* not carrying any plasmid) (Fig. 6a-c). Tobacco leaves co-expression of *VdMYB1* and *VdSTS2* were more resistant to *R. solanacearum* than those expressing only *VdSTS2* (Fig. 6a-b). Although there was no difference of the chlorophyll content between the co-expression of *VdMYB1* and *VdSTS2* leaves and expression *VdSTS2* leaves after *R. solanacearum* infection within 3 days (Fig. 6c). After 6 days, the only expression *VdSTS2* leaves showed significant lower chlorophyll content than the co-expression of *VdMYB1* and *VdSTS2* leaves (Fig. 6c). To explore the basis of this resistance, we examined the transcript level of genes involved in defense responses in transgenic and mock tobacco leaves after inoculation with *R. solanacearum*. Expression of *PR1* and *PDF1.2* did not differ between transgenic and mock tobacco leaves (Fig. 7a, b). However, co-expression of *VdMYB1* and *VdSTS2* produced higher *STS2* transcript levels and higher content of resveratrol compared with expression of *VdSTS2* alone (Fig. 7c, d). These results indicate that VdMYB1-mediated activation of *VdSTS2* increases the content of resveratrol, which enhances defense responses against pathogens.

### Transgenic tobacco plants co-expression *VdMYB1* and *VdSTS2* exhibit resistance to *R. solanacearum*

**Fig. 8:**
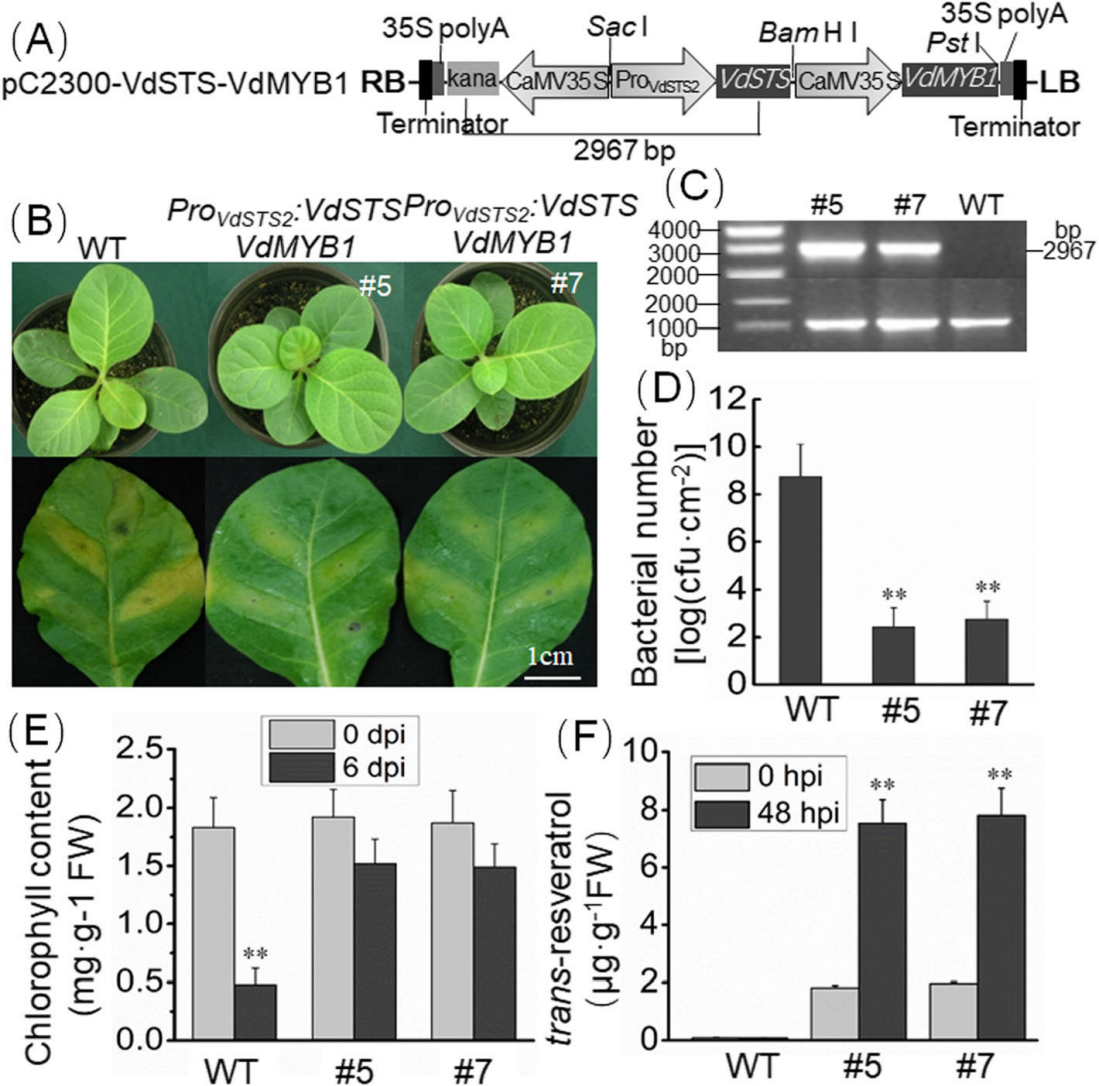
Stable transgenic tobacco plants overexpressing *VdSTS2* and *VdMYB1* show increased resistance to *R. solanacearum*. **a** Schematic representation of constructs used for tobacco transformation. B, right border; LB, left border; CaMV 35S, CaMV 35S promoter; 35S polyA, CaMV 35S polyA terminator. **b** Transgenic tobacco plants show enhanced resistance to *R. solanacearum*. The phenotype of WT and transgenic tobacco plants is shown in the top panel, and disease symptoms on the leaves of WT and transgenic lines at 6 dpi are shown in the bottom panel. **c** Identification of transgenic lines by PCR amplification of genomic DNA. **d** Growth of bacterial population in *R. solanacearum*-inoculated transgenic and WT plants at 48hpi. **e** Chlorophyll content in WT and transgenic plants at 0dpi and 6 dpi. **f** Measurement of trans-resveratrol content in WT and transgenic tobacco plants after *R. solanacearum* infection using HPLC. Error bars represent SD. Asterisks indicate statistically significant differences (**P < 0.01; Student’s *t*-test)

To confirm that VdMYB1 enhances disease resistance by activating *VdSTS2*, we generated stable transgenic tobacco lines co-expressing *VdMYB1* and *VdSTS2* (Fig. 8a) using *Agrobacterium*-mediated transformation. Transformed plants were selected by screening for kanamycin resistance and PCR. primers specific to the kanamycin selectable marker gene and *VdSTS2* coding sequence yielded a 2, 967 bp fragment in the evaluated T0 transformants (Fig. 8a, c). No similar sized PCR product was obtained from untransformed lines, thus confirming the specificity of PCR primers (Fig. 8c). Transgenic tobacco lines showed higher expression level of *VdMYB1* and *VdSTS2* than the WT (Additional file 3: Figure S2). Leaves of 7-week-old plants of two transgenic lines and WT plants were inoculated with *R. solanacearum*. At 48 hpi, *VdMYB1* and *VdSTS2* transcripts were highly upregulated in transgenic tobacco lines. At 6 dpi, WT plants showed severe wilting symptoms, whereas both transgenic lines displayed less severe disease symptoms (Fig. 8b). To evaluate the progression of disease in *R. solanacearum*-infected plants, we quantified bacterial population in infected leaves of WT and transgenic tobacco plants at 48 hpi. Significantly lower bacterial growth was observed in transgenic tobacco plants compared with WT plants (Fig. 8d). We also examined chlorophyll content in WT and transgenic leaves inoculated with *R. solanacearum*. Infection did not change the leaves chlorophyll content of the two transgenic lines (Fig. 8e). However, in WT leaves chlorophyll content showed significant difference (Fig. 8e). Additionally, measurement of resveratrol content showed that transgenic plants were able to synthesize resveratrol, unlike WT plants (Fig. 8f). After inoculation with *R. solanacearum,* the level of resveratrol significantly higher in transgenic plants than that before inoculation (Fig. 8f).

## Discussion

The MYB TFs play important roles in various the regulatory networks affecting biotic and abiotic stresses, metabolism, and plant development [9]. In grapevine, MYB TFs regulate the flavonoids biosynthesis pathway [12, 13]. However, little is known about how MYB TF mediated transcriptional regulation affects grapevine responses to pathogen. Here, we showed that VdMYB1, an R2R3-type MYB TF from the disease-resistant wild grapevine *V. davidii* cv. Tangwei, regulates the expression of the *VdSTS2* gene by directly binding to its promoter, thus contributing to enhanced resistance against pathogens.

Sequence analyses showed that the VdMYB1 protein is an R2R3-type MYB TF. VdMYB1 localized in the nuclei of *Arabidopsis* protoplasts and onion epidermal cells and functioned as a VdMYB1 transcriptional activator in yeast. Both these results are typical characteristics of a TF. Most TFs directly target a specific *cis*-element. The MYB TFs can bind to the MYBBS with the core sequence ACCTACC [20]. In grapevine leaves overexpressing *VdMYB1*, we found that the expression of key genes in the salicylic acid or jasmonate pathways did not change; however, the transcripts of *VdSTS2* gene were more abundant. This surprising finding prompted us to speculate that VdMYB1 targets the promoter of *VdSTS2* gene. Several lines of evidence collected in this study support this hypothesis. First, the *VdSTS2* promoter contains several variants of the MYBBS, such as MYB2CONSENSUSAT, MYBCORE, MYBGAHV, MYBPLANT, MYBPZM, and MYBST1. Second, overexpression of *VdMYB1* in grapevine leaves upregulated *VdSTS2* transcripts, and also resulted in increased content of resveratrol. Third, VdMYB1 activated *GUS* reporter gene expression driven by the *VdSTS2* promoter in *Arabidopsis* protoplasts GUS activity showed that the region with the *VdSTS2* promoter with the highest recognition efficiency was between − 1500 and − 500 bp. Analysis of the *VdSTS2* promoter using PLACE showed that this core region contains two classic MYBBS. Fourth, EMSAs showed that the VdMYB1 recombinant protein binds to the probe that containing an MYBBS, and the binding activity was correlated with the number of MYBBSs. Lastly, transformation of tobacco leaves showed that *STS2* transcripts are more abundant and produce higher contents of resveratrol in leaves co-transformed with *VdMYB1* and *STS2* under its own promoter.

In grapevine, MYB TFs participate in the regulation of the flavonoid biosynthetic pathway. In cultivated grapevine (*V. vinifera*), VviMYB5a and VviMYB5b are two homologous R2R3-type MYB TFs that regulate flavonoid biosynthesis [11, 21]. The MYB TFs VviMYBPA1 and VviMYBPA2 also act during seed development for regulating the proanthocyanidin pathway [22, 23]. Another R2R3-type MYB TF,VviMYBF1 regulates the biosynthesis of flavonols [24]. However, there is little information showing that MYB TFs act in defense responses in grapevine. By contrast, MYB TFs in *Arabidopsis* and other plants have been shown to play important roles in defense responses [7]. In *Arabidopsis*, the expression of BOS1, an R2R3-type MYB TF, is significantly induced following infection with *Botrytis cinerea* [25], and the *bos1* mutant exhibits increased susceptibility to *B. cinerea*, *Alternaria brassicicola*, *Pseudomonas syringae* pv *tomato*, and *Peronospora parasitica* [25]. Another R2R3-type MYB-related protein and a putative TF in *Arabidopsis thaliana*, MYB30, positively regulates programmed cell death associated with hypersensitive response [26]. In this study, we showed that transcript levels of *VdMYB1* were significantly increased after inoculation with the PM fungus, *E. necator*. Transient overexpression of *VdMYB1* in grapevine leaves resulted in resistance to PM and the production of abundant ROS within 38 min of infection. More recent studies showed that SA promoted the biosynthesis of flavan-3-ol and PAs in Poplar by activating the MYB-bHLHWD40 complex against rust proliferation and infection [27]. The present finding also support author study which concluded that SA increased flavonoid accumulation in plant by inducing MYB TFs.

The STS enzyme participates in the biosynthesis of stilbenes, which function as antimicrobial compounds. Expression of *VdMYB1* in grapevine leaves led to the accumulation of more *STS* transcripts and production of high levels of resveratrol. Moreover, infection of *VdMYB1*-overexpressing grapevine leaves with PM resulted in significantly higher *STS* compared with mock. Furthermore, transient expression of the *VdSTS2* under the control of its native promoter showed that the transformed tobacco leaves were more resistant to *R. solanacearum* compared with the mock. The resistance to *R. solanacearum* was even higher in leaves co transformed with *VdMYB1* and *VdSTS2*. Reactive oxygen species (ROS) play a central role in plant defense against various pathogens [28]. By analyzing the ROS accumulation in *VdMYB1*-expressing plants, we found that the contents of H_2_O_2_ were induced upon PM infection, while the control plants were maintained at lower levels. These findings indicate that *VdMYB1* may play a role in the regulation of ROS production. ROS was accumulated at the pathogen attack site in plants, which called oxidative burst, is can lead to a hypersensitive response (HR) that prevents biotrophic pathogens further spread [29]. Recent research has indicated that ROS not only function as toxic compounds that lead HR, but also act important early signal molecules [30]. Duan et al., proposed that ROS are necessary for the induction of stilbene synthase, and resulted in production of proanthocyanidin [19]. Jiao et al., reported that the accumulation of trans-resveratrol, trans-piceid and cis-piceid markedly increased after treatment with H_2_O_2_ [31]. We proposed that overexpression *VdMYB1* resulted in plant accumulation ROS to prevent pathogen invasion.

Most MYB TFs directly target the promoters of downstream genes. TRANSPARENT TESTA 2 (TT2), an *Arabidopsis* R2R3 MYB domain protein, provides target specificity to the MYB-bHLH-WD complex, which activates various phytoalexin biosynthetic genes, including *DFR*, *TT12*, *AHA10,* and *BAN* [32]. The MYB TF MYB134 in poplar shows strong sequence similarity to *Arabidopsis* TT2, and overexpression of *MYB134* activates the phytoalexin biosynthesis pathway and significantly increase phytoalexin levels in poplar [10]. Results of EMSA show that recombinant MYB134 protein binds to the promoter regions of genes in the phytoalexin pathway [33]. In grapevine, VviMYB5a and VviMYB5b activate the expression of *VviCHI*, *VviF3’5’H*, and *VviANS*, which encode enzymes of the general flavonoid biosynthesis pathway and participate in synthesis of phytoalexins, anthocyanins, and flavonols, respectively [12, 21]. In addition, VviMYB5b activates the expression of *VviLAR1* and *VviANR*, whereas VviMYB5a is implicated only in the regulation of *VviLAR1* expression [12, 13]. Two MYB proteins in grapevine, VviMybPA1 and VviMybPA2, appear to be the closest orthologues of *Arabidopsis* TT2 [22, 23]. VviMybPA1 or VviMybPA2 significantly upregulate the expression of genes encoding enzymes of the flavonoid biosynthesis pathway, including ANR and LAR1 [22, 23]. Additionally, VviMYBF1 activates the expression of the flavonol-specific gene *VviFLS1* but not that of *VviUFGT* or *VviANR* [25]. The MYB TF VviMYB14 and its homolog VviMYB15 co-express with *VviSTS* in leaf tissues under biotic and abiotic stresses and in berries during maturation [34]. Transient expression of *VviMYB14* and *VviMYB15* results in enhanced transcript levels of *VviSTS* [34]. In the grapevine hairy root system, overexpression of *VviMYB15* results in higher *VviSTS* transcript levels and the accumulation of glycosylated stilbenes [34].

## Conclusion

Together, these data confirm that VdMYB1 TFs regulate the stilbenoids biosynthesis pathway at the transcriptionally level. Our results showed that VdMYB1 binds to the *VdSTS2* promoter both in vivo and in vitro, and overexpression of *VdMYB1* in grapevine and tobacco leaves resulted in increased accumulation of *VdSTS2* transcripts. High levels of *VdSTS2* transcripts, and consequently increase in resveratrol content, resulted in greater resistance to *E. necator* in grapevine leaves and to *R. solanacearum* in tobacco leaves.

## Methods

### Plant materials

*V. davidii* cv. Tangwei were cultivated in the experimental vineyard of the germplasm repository for grapes in the Institute of Botany, Chinese Academy of Sciences in Beijing.

### RNA extraction and VdMYB1 cDNA isolation

Total RNA was extracted from the leaves of *V. davidii* cv. Tangwei, as described previously by Yu et al. [35]. Total RNA was treated with RNase-free DNase I for 30 min to remove traces of contaminating genomic DNA, and used for cDNA synthesis using SMARTer RACE cDNA Amplification Kit (Clontech, Japan), according to the manufacturer’s instructions. The *VdMYB* cDNA was isolated by PCR using degenerate primers designed based on the partial *MYB* sequence of *V. vinifera* and Ex Taq HS DNA polymerase (Takara, Dalian, China). Subsequently, the sequence of the amplified partial cDNA fragment was used for designing gene specific primers (Additional file 1: Table S1), which were used for performing RACE PCR according to the manufacturer’s instructions. PCR products obtained from all successful amplifications were cloned into the using pGEM-T Easy vector (Promega, Beijing, China) and five independent clones has sequenced.

### qRT-PCR

Total RNA was extracted from treated grapevine leaf and all tissues as described above. To generate first-strand cDNA, 0.5 μg of DNase I-treated total RNA was reverse transcribed in 10 μL volume using PrimeScript Reverse Transcriptase Kit according to the manufacturer’s instructions (TaKaRa, Dalian, China). Subsequently, qRT-PCR was performed on a Bio-Rad IQ^5^ Real-Time PCR Detection System (Bio-Rad Laboratories, Hercules, CA, USA) using SYBR Premix Ex Taq II, according to the manufacturer’s instructions (TaKaRa, Dalian, China). Primers used for qRT-PCR are listed in Additional file 1: Table S1. PCR reactions were prepared in 96-well plates (Bio-Rad, USA), and each reaction contained 1 μL of diluted cDNA (100 ng/μL), 12.5 μL of SYBR Green PCR Master Mix, 1 μL of each primer (at 250 nM) in a final volume of 25 μL. The qRT-PCR was performed using the following conditions: initial denaturation at 94 °C for 3 min, followed by 40 cycles of denaturation at 94 °C for 15 s, annealing at 58 °C for 30 s, and extension at 72 °C for 30 s, and lastly melting curve analysis at 60–94 °C. The *VdGAPDH* gene was used as a reference gene for data normalization according to the 2^-ΔΔc(t)^ method [36]. All reactions were conducted in three technical replicates. Mean values of three independent experiments were analyzed using the Student’s *t*-test.

### Subcellular localization and transcriptional activation assays

The *VdMYB1* ORF minus the stop codon was cloned into the pBI221-GFP vector using *Xba*I and *Kpn*I restriction sites. In the resulting vector pBI221-GFP/VdMYB1, the GFP-VdMYB1 fusion was driven by the CaMV 35S promoter. Primers used for cloning are listed in Additional file 1: Table S1. Sequence analysis was used to verify the final construct. Gene gun bombardment and polyethylene glycol (PEG) transformation methods were used to introduce the constructs (including the vector-only negative control) into onion epidermal cells and *Arabidopsis* protoplasts as described previously [36]. Transformed cells were incubated in a growth chamber at 24 °C for 16–18 h. GFP signals were detected by confocal laser scanning microscopy (Zeiss LSM 510, Oberkochen, Germany). At least three replicates were performed for all transformations.

To generate the pGBKT7/VdMYB1 fusion construct, the *VdMYB1* ORF minus the stop codon was cloned into the pGBKT7 vector using *Nde*I and *Bam*H I restriction site. Full-length *GAL4* gene sequence from the pCL1 plasmid was cloned into the pGBKT7 vector to serve as the positive control (pGBKT7/GAL4), and the pGBKT7 vector was used as the negative control. Primers used for PCR are listed in Additional file 1: Table S1. The final constructs were verified by sequencing. The control plasmid and the pGBKT7/VdMYB1 were transformed into yeast (strain AH109). After transformation, yeast cells were cultured on SD/−Trp medium at 28 °C for 3 days. Three transformants were then cultured on SD/−His/−Ade/−Trp medium with X-α-gal at 28 °C for 3 days.

### *Agrobacterium*-mediated transient expression assay

The *VdMYB1* ORF was cloned into pBI121 binary vector to generate the construct pBI121/VdMYB1 for transient expression assay. The *VdSTS*2 promoter sequence (− 1500 to 0) was amplified from genomic DNA. A series of nested 5′ deletions of the *VdSTS2* promoter was generated by PCR using sequence-specific primers (Additional file 1: Table S1). Three *STS* promoter sequences (*ProSTSful*, *ProSTSdel1*, and *ProSTSdel2*) were cloned into the pC0390GUS vector using *Pst*I and *Eco*RI restriction site to generate *ProSTSful::GUS*, *ProSTSdel1::GUS*, and *ProSTSdel2::GUS*, respectively. The recombinant plasmid was confirmed by sequencing. All constructs were transformed into *Agrobacterium tumefaciens* strain GV3101 by electroporation. Leaves of 8-week-old *V. vinifera* cv. Carignane plants or 6-week-old tobacco plants in vitro were detached and used for transformation, according to the methods described by Xu [37] and Sparkes [38]. GUS activity was measured as described previously [38, 39], and each GUS assay was performed at least three times. The amount of ROS was measured using a luminol-based assay, as described by Keppler et al. [40]. Resveratrol in tobacco leaves was measured by HPLC analysis. Total chlorophyll was extracted using 80% acetone and measured at 645 nm and 663 nm absorbance.

### *Agrobacterium*-mediated transformation of tobacco plants

The Pro_VdSTS2_:VdSTS2 cassette containing *Sac*I and *Bam*HI restriction sites was ligated into pCAMBIA2300 binary vector, to generate the construct pC2300/VdSTS2. The VdMYB1 ORF was cloned into pC2300/VdSTS2, to generate the stable transformation vector pC2300-VdSTS2-VdMYB1. For tobacco transformation, the binary vector pC2300-VdSTS2-VdMYB1 was introduced into *Agrobacterium* strain LBA4404. Leaf discs (8 mm diameter) collected from fully expanded leaves of tobacco plants were used for *Agrobacterium*-mediated transformation. Putative transformants were screened by PCR using genomic DNA isolated from WT plants and various transgenic lines as template.

### EMSAs

All five MYBBSs in the *VdSTS* promoter sequence (− 1400 to − 590) were mutated using the MutanBEST Kit (Takara, Dalian, China) to generate MYBBSm1–5 mutant fragments. To perform EMSAs, MYBBS and MYBBSm1–5 probe fragments were obtained by PCR amplification and labeled with [γ-^32^P] ATP using T4 polynucleotide kinase (New England Biolabs, Hitchin, UK). The *VdMYB1* ORF was cloned into the pGEX-6P-1 vector, and transformed into *Escherichia. coli* strain BL21(DE3). IPTG was added to the bacterial culture to induce expression of the GST-VdMYB1 fusion protein. The expressed fusion proteins were purified using GST SpinTrap columns (GE Healthcare). Labeled probes were incubated with 50 ng of GST-VdMYB1 in binding buffer (10 mM Tris [pH 7.5], 5 mM MgCl_2_, 50 mM KCl, 100 μg/mL BSA, 2.5% glycerol, 1 mM DTT, and 50 ng/μL poly (dI-dC) for 25 min. The fusion protein-bound probes were separated from unbound probes using PAGE (5%). Gels were dried, and signal was detected by overnight exposure to X-ray film (Kodak).

### Yeast one-hybrid assay

The yeast one-hybrid assay was performed using MATCHMAKER Gold Yeast One-Hybrid Library Screening System (Clontech) and YEASTMAKER Yeast Transformation System 2 (Clontech). The amplified promoter regions were cloned upstream of the Aureobasidin A resistance (*AUR1-C*) reporter gene in the pAbAi vector to generate the following constructs: pAbAi-proVdSTS2ful, pAbAi-proVdSTS2del1, pAbAi-proVdSTS2del2. The *VdMYB1* ORF was cloned in frame after the GAL4 transcriptional activation domain (AD) in pGADT7, and the resulting AD-VdMYB1 was co-introduced with other pAbAi vectors into the yeast strain Y1HGold. The transformed yeast cells were cultured on SD medium containing 0 or 200 ng/ml Aureobasidin A at 30 °C for 3 days. The *β*-galactosidase activity of positive clones was identified according to the manufacturer’s instructions.

### Determination of *Trans*-resveratrol content by HPLC method

Trans-resveratrol levels in berry skins and leaves were measured using HPLC method. The standard for trans-resveratrol was purchased from Sigma-Aldrich (USA). Mocked leaves and *VdMYB* overexpressed leaves inoculated with *E. necator* for 0 and 24 h. One gram samples were ground to powder using a porcelain mortar and pestle in liquid nitrogen, extracted by 15 mL extraction solution (methanol: ethyl acetate, 1:1 v/v) for 24 h at room temperature in the dark. After centrifugation at 20000 *g* at 4 °C for 10 min, the supernatant was evaporated at 40 °C until the solvent was volatilized completely and then dissolved in 2 ml methanol. The extract was filtered through a 0.22 μm PTFE membrane filter before resveratrol analysis. Extractable amounts of resveratrol were analyzed using a Waters e2695 HPLC system (USA). Elution was carried out with a mobile phase delivered using a Waters C18 HPLC pump at a flow rate 0.8 mL·min^− 1^. A Waters 2996 UV detector was used at 306 nm. Mean values and standard deviations were obtained from three biological replicates.

### Determination of chlorophyll content

Leaf tissues were ground in liquid nitrogen and 100 mg of leaf powder was used to extract pigments using 80% acetone. Chlorophyll absorbance was analyzed by spectrophotometry at 645 and 663 nm.

### Pathogen inoculation and SA, MeJA treatments

The PM pathogen *E. necator* was maintained on *V. vinifera* cv. Carignane seedlings. The inoculation of *V. davidii* cv. Tangwei leaves with *E. necator* was performed as described previously [41]. To monitor the growth of *E. necator*, conidiophores were counted as described previously [36]. *Ralstonia solanacearum* strain BJ1057 was cultured in tetrazolium agar medium at 28 °C. A needleless hypodermic syringe was used to infiltrate bacterial suspensions (10^4^ cfu/ml) into leaves of intact tobacco plants. The growth of *R, solanacearum* was measured at 5 dpi [35]. Mean values of three independent experiments were used for statistical analysis using Student’s *t*-test.

For exogenous chemical compounds treatments, 100 μM SA, and 0.5 g/L MeJA were sprayed on the selected grapevine leaves according to the method described [42], and was repeated three times on three independent plants. Leaves sprayed with sterile water were used as negative controls. After 0, 12, 24, 36, and 48 h of inoculation, the treated grapevine leaves were sampled, immediately frozen in liquid nitrogen, and stored at − 80 °C for further study.

### Bioinformatics analysis

Nucleotide sequence and putative amino acid sequence were analyzed with the basic local alignment search tool (BLAST) at the National Center for Biotechnology Information (http://www.ncbi.nlm.nih.gov) and Grape Genome website (http://www.genoscope.cns.fr/externe/GenomeBrowser/Vitis/, V2 version). Phylogenetic tree was constructed using neighbor joining method with the bootstrap values of 1000 by MEGAX. Promoter analysis was performed using online analysis software of PLACE and PlantCARE. Protein domain analysis was done by the online software of SMART (http://smart.embl-heidelberg.de/) and ExPASy (https://www.expasy.org/).

## Additional files

Additional file 1: **Table S1.** The sequences of the primers used in these experiments. (DOCX 15 kb)

Additional file 2: **Figure S1.** Relative expression level of VdMYB1 in different VdMYB1-overexpressing transgenic lines. (DOCX 555 kb)

Additional file 3: **Figure S2.** *VdSTS2* and *VdMYB1* expression level in stable transgenic tobacco plants co-overexpressing *VdSTS* and *VdMYB1*. (DOCX 377 kb)

## Data Availability

All data generated and analyzed during this study are included in this published article.

## Abbreviations

AHA10: Auto-inhibited H^+^-ATPase isoform 10
*ANR*: Anthocyanidin reductase
ANS: Anthocyanin synthase
CaMV 35S: Cauliflower mosaic virus 35 s promoter
*CHI*: Chitinases
*DFR*: Dihydroflavonol 4-reductase
EMSA: Electrophoretic mobility shift assay
*F3’5’H*: Flavonoid-3′,5′-hydroxylase
FLS1: Flavonol synthase 1
GFP: Green fluorescent protein
*GUS*: *β*-glucuronidase
LAR1: Leucoanthocyanidin reductase 1
MYBBS: MYB binding site
NAC: NAM, ATAF, and CUC
ORF: Open reading frame
*PDF1.2*: Plant defensin 1.2
PR: Pathogenesis-related
RACE: Rapid amplification of cDNA ends
ROS: Reactive oxygen species
SANT: SWI3, ADA2, N-CoR, and TFIIIB domains
SMART: Simple modular architecture research tool
STS: Stilbene synthases
TFs: Transcription factors
TT2: TRANSPARENT TESTA 2
*UFGT*: Flavonoid 3-O-glucosyltransferase
VdMYB1: *Vitis davidii* MYB1

## References

[1] Jones JD, Dangl JL (2006). The plant immune system. Nature.

[2] Rushton PJ, Somssich IE (1998). Transcriptional control of plant genes responsive to pathogens. Curr Opin Plant Biol.

[3] Kesarwani M, Yoo J, Dong X (2007). Genetic interactions of TGA transcription factors in the regulation of pathogenesis-related genes and disease resistance in Arabidopsis. Plant Physiol.

[4] Fujimoto SY, Ohta M, Usui A, Shinshi H, Ohme-Takagi M (2000). Arabidopsis ethylene-responsive element binding factors act as transcriptional activators or repressors of GCC box–mediated gene expression. Plant Cell.

[5] Desveaux D, Subramaniam R, Després C, Mess J-N, Lévesque C, Fobert PR, Dangl JL, Brisson N (2004). A “whirly” transcription factor is required for salicylic acid-dependent disease resistance in Arabidopsis. Dev Cell.

[6] Fujita M, Fujita Y, Maruyama K, Seki M, Hiratsu K, Ohme-Takagi M, Tran LSP, Yamaguchi-Shinozaki K, Shinozaki K (2004). A dehydration-induced NAC protein, RD26, is involved in a novel ABA-dependent stress-signaling pathway. Plant J.

[7] Dubos C, Stracke R, Grotewold E, Weisshaar B, Martin C, Lepiniec LJ (2010). MYB transcription factors in Arabidopsis. Trends Plant Sci.

[8] Chen Y, Yang X, He K, Liu M, Li J, Gao Z, Lin Z, Zhang Y, Wang X, Qiu X, Shen Y, Zhang L, Deng X, Luo J, Deng X (2006). The MYB transcription factor superfamily of Arabidopsis: expression analysis and phylogenetic comparison with the rice MYB family. Plant Mol Biol.

[9] Chaonan L, Ng CK-Y, Fan L (2015). MYB transcription factors, active players in abiotic stress signaling. Environ Exp Bot.

[10] Ullah C, Unsicker SB, Fellenberg C, Constabel CP, Schmidt A, Gershenzon J, Hammerbacher A (2017). Flavan-3-ols are an effective chemical defense against rust infection. Plant Physiol.

[11] Cavallini E, Matus JT, Finezzo L, Zenoni S, Loyola R, Guzzo F, Schlechter R, Ageorges A, Arce-Johnson P, Tornielli GB (2015). The phenylpropanoid pathway is controlled at different branches by a set of R2R3-MYB C2 repressors in grapevine. Plant Physiol.

[12] Deluc L, Barrieu F, Marchive C, Lauvergeat V, Decendit A, Richard T, Carde J-P, Mérillon J-M, Hamdi S (2006). Characterization of a grapevine R2R3-MYB transcription factor that regulates the phenylpropanoid pathway. Plant Physiol.

[13] Koyama K, Numata M, Nakajima I, Goto-Yamamoto N, Matsumura H, Tanaka N (2014). Functional characterization of a new grapevine MYB transcription factor and regulation of proanthocyanidin biosynthesis in grapes. J Exp Bot.

[14] Guo D, Wang Z, Li Q, Gu S, Zhang G, Yu Y (2019). Hydrogen peroxide treatment promotes early ripening of Kyoho grape. Aust J Grape Wine Res.

[15] Guo D, Zhao H, Li Q, Zhang G, Jiang J, Liu C, Yu Y (2019). Genome-wide association study of berry-related traits in grape based on genotyping-by-sequencing markers. Hortic Res.

[16] Yu Y, Jiao Z, Bian L, Wan Y, Yu K, Zhang G, Guo D (2019). Overexpression of Vitis vinifera VvbZIP60 enhances Arabidopsis resistance to powdery mildew via the salicylic acid signaling pathway. Sci Hortic.

[17] Wang Y, Liu Y, He P, Chen J, Lamikanra O, Lu J (2015). Evaluation of foliar resistance to *Uncinula necator* in Chinese wild Vitis species. Vitis.

[18] Kumar S, Stecher G, Li M, Knyaz C, Tamura K (2018). MEGA X: molecular evolutionary genetics analysis across computing platforms. Mol Biol Evol.

[19] Bai R, Luo Y, Wang L, Li J, Wu K, Zhao G, Duan D (2019). A specific allele of MYB14 in grapevine correlates with high stilbene inducibility triggered by Al3+ and UV-C radiation. Plant Cell Rep.

[20] Shen H, He X, Poovaiah CR, Wuddineh WA, Ma J, Mann DG, Wang H, Jackson L, Tang Y, Neal Stewart C (2012). Functional characterization of the switchgrass (Panicum virgatum) R2R3-MYB transcription factor PvMYB4 for improvement of lignocellulosic feedstocks. New Phytol.

[21] Deluc L, Bogs J, Walker AR, Ferrier T, Decendit A, Merillon J-M, Robinson SP, Barrieu F (2008). The transcription factor VvMYB5b contributes to the regulation of anthocyanin and proanthocyanidin biosynthesis in developing grape berries. Plant Physiol.

[22] Bogs J, Jaffé FW, Takos AM, Walker AR, Robinson SP (2007). The grapevine transcription factor VvMYBPA1 regulates proanthocyanidin synthesis during fruit development. Plant Physiol.

[23] Terrier N, Torregrosa L, Ageorges A, Vialet S, Verriès C, Cheynier V, Romieu C (2009). Ectopic expression of VvMybPA2 promotes proanthocyanidin biosynthesis in grapevine and suggests additional targets in the pathway. Plant Physiol.

[24] Czemmel S, Stracke R, Weisshaar B, Cordon N, Harris NN, Walker AR, Robinson SP, Bogs J (2009). The grapevine R2R3-MYB transcription factor VvMYBF1 regulates flavonol synthesis in developing grape berries. Plant Physiol.

[25] Mengiste T, Chen X, Salmeron J, Dietrich R (2003). The BOTRYTIS SUSCEPTIBLE1 gene encodes an R2R3MYB transcription factor protein that is required for biotic and abiotic stress responses in Arabidopsis. Plant Cell.

[26] Marino D, Froidure S, Canonne J, Khaled SB, Khafif M, Pouzet C, Jauneau A, Roby D, Rivas S (2013). Arabidopsis ubiquitin ligase MIEL1 mediates degradation of the transcription factor MYB30 weakening plant defence. Nat Commun.

[27] Ullah C, Tsai CJ, Unsicker SB, Xue L, Reichelt M, Gershenzon J, Hammerbacher A (2019). Salicylic acid activates poplar defense against the biotrophic rust fungus Melampsora larici-populina via increased biosynthesis of catechin and proanthocyanidins. New Phytol.

[28] Liu X, Williams CE, Nemacheck JA, Wang H, Subramanyam S, Zheng C, Chen M-S (2010). Reactive oxygen species are involved in plant defense against a *gall midge*. Plant Physiol.

[29] Torres MA, Jones JD, Dangl JL (2006). Reactive oxygen species signaling in response to pathogens. Plant Physiol.

[30] Miller G, Coutu J, Shulaev V, Mittler R. Reactive oxygen signalling in plants. In Intracellular Signalling in Plants (ed. Z. Yang) Annual Plant Reviews, vol. 33, pp. 189–201. Oxford: WileyBlackwell; 2008.

[31] Jiao Y, Wang D, Wang L, Jiang C, Wang Y (2017). VqMAPKKK38 is essential for stilbene accumulation in grapevine. Hortic Res.

[32] Lepiniec L, Debeaujon I, Routaboul J-M, Baudry A, Pourcel L, Nesi N, Caboche M (2006). Genetics and biochemistry of seed flavonoids. Annu Rev Plant Biol.

[33] Mellway RD, Tran LT, Prouse MB, Campbell MM, Constabel CP (2009). The wound-, pathogen-, and ultraviolet B-responsive MYB134 gene encodes an R2R3 MYB transcription factor that regulates proanthocyanidin synthesis in poplar. Plant Physiol.

[34] Höll J, Vannozzi A, Czemmel S, D'Onofrio C, Walker AR, Rausch T, Lucchin M, Boss PK, Dry IB, Bogs J (2013). The R2R3-MYB transcription factors MYB14 and MYB15 regulate stilbene biosynthesis in *Vitis vinifera*. Plant Cell.

[35] Yu Y, Xu W, Wang J, Wang L, Yao W, Yang Y, Xu Y, Ma F, Du Y, Wang Y (2013). The Chinese wild grapevine (*Vitis pseudoreticulata*) E3 ubiquitin ligase Erysiphe necator-induced RING finger protein 1 (EIRP1) activates plant defense responses by inducing proteolysis of the VpWRKY11 transcription factor. New Phytol.

[36] Livak KJ, Schmittgen TD (2001). Analysis of relative gene expression data using real-time quantitative PCR and the 2^− ΔΔCT^ method. Methods.

[37] Xu W, Yu Y, Ding J, Hua Z, Wang Y (2010). Characterization of a novel stilbene synthase promoter involved in pathogen-and stress-inducible expression from Chinese wild *Vitis pseudoreticulata*. Planta.

[38] Jefferson RA (1987). Assaying chimeric genes in plants: the GUS gene fusion system. Plant Mol Biol Report.

[39] Bradford MM (1976). A rapid and sensitive method for the quantitation of microgram quantities of protein utilizing the principle of protein-dye binding. Anal Biochem.

[40] Keppler LD, Baker CJ, Atkinson MM (1989). Active oxygen production during a bacteria-induced hypersensitive reaction in tobacco suspension cells. Phytopathology.

[41] Sparkes IA, Runions J, Kearns A, Hawes C (2006). Rapid, transient expression of fluorescent fusion proteins in tobacco plants and generation of stably transformed plants. Nat Protoc.

[42] Li H, Xu Y, Xiao Y, Zhu Z, Xie X, Zhao H, Wang YJ (2010). Expression and functional analysis of two genes encoding transcription factors, VpWRKY1 and VpWRKY2, isolated from Chinese wild *Vitis pseudoreticulata*. Plant Mol Biol Report.

