# Supplementary material for: The grapevine R2R3-type MYB transcription factor VdMYB1 positively regulates defense responses by activating the stilbene synthase gene 2 (VdSTS2)
Source: BMC Plant Biol. 2019 Nov 7;19:478. doi: 10.1186/s12870-019-1993-6 (PMC6836392; doi:10.1186/s12870-019-1993-6)
Supplement: Supplementary file 1 — Table S1. The sequences of the primers used in these experiments. (DOCX 15 kb) [file 12870_2019_1993_MOESM1_ESM.docx]

Table S1. The sequences of the primers used in these experiments

| Primer name | oligonucleotide primers | Purpose or vector |
| --- | --- | --- |
| VdMYB1-F  VdMYB1-R | AAAGGCTGGGCAATAGATGG  TGCAGGGTGTAGTAATGTCGG | PCR |
| VdMYB1-5’race | GTCCTCCCCGGTAGTTTCGCTGCTATCGCTG | RACE-PCR |
| VdMYB1-3’race | GGACCAGTACTGTCCCCACAGCCGTCC | RACE-PCR |
| VdMYB1qt-F  VdMYB1qt-R | TATGAGGGAAGGGGCTTGTAA  TCATTTGGCTGAGTCTGTTCG | qtRT-PCR |
| VdGAPDH-F  VdGAPDH-R | CCCTTGTCCTCCCAACTCT  CCTTCTCAGCACTGTCCCT | qtRT-PCR |
| VdPR1-F  VdPR1-R | GGAGTCCATTAGCACTCCTTTG  CATAATTCTGGGCGTAGGCAG | qtRT-PCR |
| VdPDF1.2-F  VdPDF1.2-R | CGTCGATGCTTCTGCACTAAG  TAGACCGCAGATCAAACCAAA | qtRT-PCR |
| VdSTS-F  VdSTS-R | AGAGTGGGGTCAGCCTAAATC  CCAACTAAAGAGTCCAAAGCATC | qtRT-PCR |
| NtPR1-F  NtPR1-R | TAGTCATGGGATTTGTTCTC  TCAGATCATACATCAAGCTG | qtRT-PCR |
| NtPDF1.2-F  NtPDF1.2-R | TTGCTTGTCACGGCTAC  ACCGAAATTGGATACCTT | qtRT-PCR |
| NtEF1α-F  NtEF1α-R | AAGTATGCCTGGGTGCTTG  AGGGACAGTACCAATTCCACC | qtRT-PCR |
| VdMYB1-XbaI-F  VdMYB1-KpnI-R | GGCTCTAGAATGGGGAGAGCTCCTTGTTGTGA  GCGGGTACCCAAGCCCCTTCCCTCATATTTCTG | pBI221-GFP |
| VdMYB1-NdeI-F  VdMYB1-BamHI-R | GGGCATATGATGGGGAGAGCTCCTTGTTGTGA  GGGGGATCCCAAGCCCCTTCCCTCATATTTCTG | pGBKT7 |
| VdSTSPF-Pst I-F  VdSTSPF-EcorR I | TTGGTTCTGCAGCTAATTACATCTAATAATGATAGGATATAAGCATTTG  GGGGAATTCATTGATCCCAGCTACGTACTCAAATTAAAGCCTAA | *ProSTSful::GUS* |
| VdSTSPd1-Pst I-F  VdSTSPF-EcorR I | TTGGTTCTGCAGTTATAAGGACTTTATAGTGCCTTTTTGGAAAGAAAC  GGGGAATTCATTGATCCCAGCTACGTACTCAAATTAAAGCCTAA | *ProSTSdel1::GUS* |
| VdSTSPd2-Pst I-F  VdSTSPF-EcorR I | TTGGTTCTGCAGTAAGTTAAATAACATGTGATGTAATCCTGCAAAAGA  GGGGAATTCATTGATCCCAGCTACGTACTCAAATTAAAGCCTAA | *ProSTSdel2::GUS* |
