# Supplementary material for: The grapevine R2R3-type MYB transcription factor VdMYB1 positively regulates defense responses by activating the stilbene synthase gene 2 (VdSTS2)
Source: BMC Plant Biol. 2019 Nov 7;19:478. doi: 10.1186/s12870-019-1993-6 (PMC6836392; doi:10.1186/s12870-019-1993-6)
Supplement: Supplementary file 2 — Figure S1. Relative expression level of VdMYB1 in different VdMYB1-overexpressing transgenic lines. (DOCX 555 kb) [file 12870_2019_1993_MOESM2_ESM.docx]

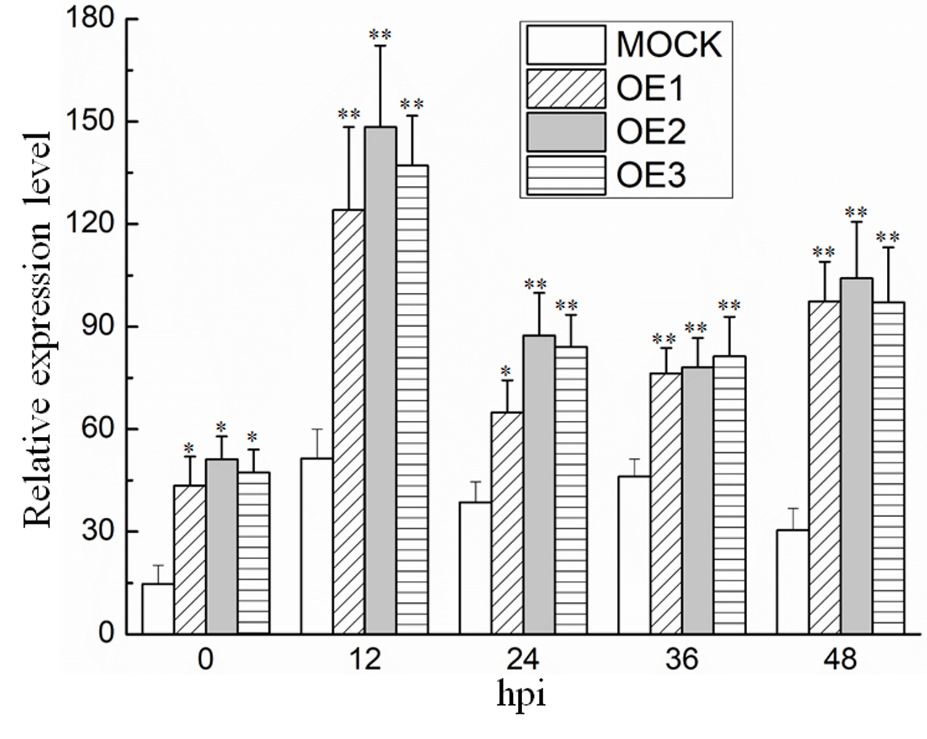


**Figure S1. Relative expression level of *VdMYB1* in different *VdMYB1*-overexpressing transgenic lines.**

After 24 h transient expression *VdMYB1* by *Agrobacterium*-mediated transformation, transgenic and mock leaves were inoculated with *Ralstonia solanacearum*. Error bars represent the SD. Asterisks indicate significant differences by Student’s *t-*test (**P* < 0.05, ***P* < 0.01).
