# Supplementary material for: The grapevine R2R3-type MYB transcription factor VdMYB1 positively regulates defense responses by activating the stilbene synthase gene 2 (VdSTS2)
Source: BMC Plant Biol. 2019 Nov 7;19:478. doi: 10.1186/s12870-019-1993-6 (PMC6836392; doi:10.1186/s12870-019-1993-6)
Supplement: Supplementary file 3 — Figure S2. VdSTS2 and VdMYB1 expression level in stable transgenic tobacco plants co-overexpressing VdSTS and VdMYB1. (DOCX 377 kb) [file 12870_2019_1993_MOESM3_ESM.docx]

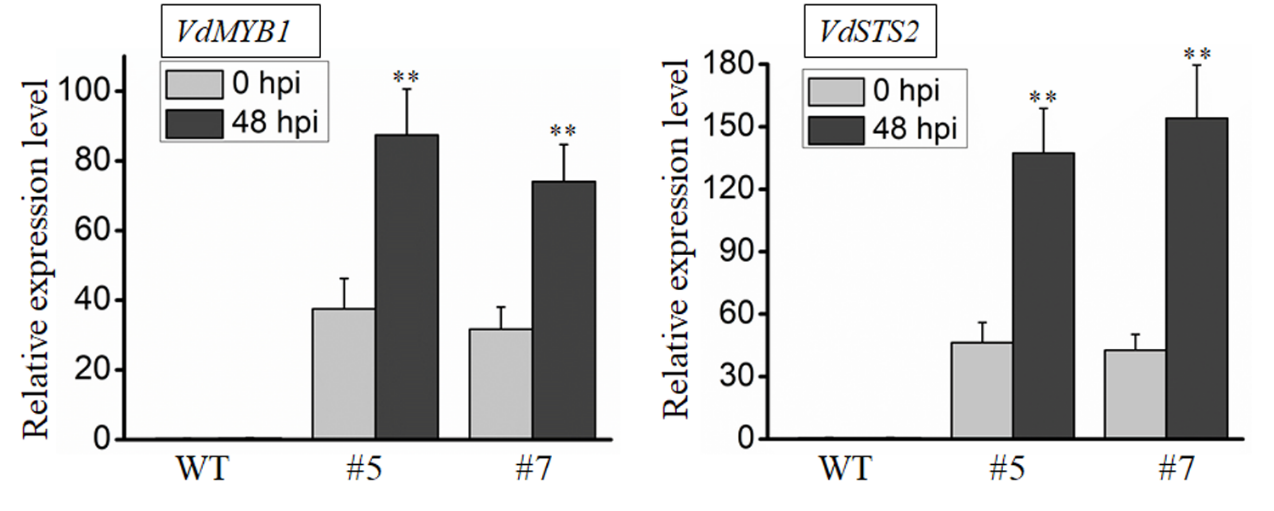


**Figure S2. *VdSTS2* and *VdMYB1* expression level in stable transgenic tobacco plants co-overexpressing *VdSTS* and *VdMYB1***

Seven-week-old plants of the two transgenic lines and WT plants were inoculated in leaves with *R. solanacearum*. Error bars represent the SD. Asterisks indicate significant differences by Student’s t-test (**P* < 0.05, ***P* < 0.01).
